# Extrudability and Mechanical Properties of Wood–Sodium Silicate Composites with Hemp Fiber Reinforcement for Additive Manufacturing

**DOI:** 10.3390/polym17182478

**Published:** 2025-09-13

**Authors:** Nagendra G. Tanikella, Alexandra M. Lehman-Chong, Armando G. McDonald, Michael R. Maughan

**Affiliations:** 1 Department of Mechanical Engineering, University of Idaho, Moscow, ID 83844, USA; tani7947@vandals.uidaho.edu (N.G.T.); a.lehman-chong@wsu.edu (A.M.L.-C.); 2 Voiland School of Chemical Engineering and Bioengineering, Washington State University, Pullman, WA 99164, USA; 3 Department of Forest, Rangeland, and Fire Sciences, University of Idaho, Moscow, ID 83844, USA; armandm@uidaho.edu

**Keywords:** hemp fiber, fiber-reinforced composites, thermosets, additive manufacturing, wood fiber

## Abstract

This study investigates the potential of hemp fiber reinforcement in wood–sodium silicate composites for additive manufacturing. It focuses on the impact of hemp fiber length and content on the rheological, flexural, compression properties, and extrudability of the composite. Composites contained varying amounts of sodium silicate (45, 50, 55 wt%) and hemp fibers of varying lengths (1, 3, 5 mm) and amounts (2.5, 5, 10 wt%) along with wood fibers sifted through a 40-mesh sieve. The study shows that higher sodium silicate content significantly increases viscosity while reducing the motor power needed to extrude the composite. Hemp fiber amount positively affects flexural and compression strength, increasing by 31.2% and 35.6%, respectively, with 5 wt% hemp fiber. This improvement in mechanical properties significantly increases the thermoset-based composite’s potential for various applications. This study also demonstrates for the first time, the feasibility of using the hemp fiber-reinforced wood–sodium silicate composite for additive manufacturing by successfully depositing a multi-layer sample print and determining its bending strength.

## 1. Introduction

Approximately 83.4 million tons of primary wood and bark processing residues were generated in 2010 [1]. The growing global focus on sustainability has led to significant advancements in the development of wood-based composites. This approach not only addresses environmental concerns but also offers an efficient way to repurpose wood residues associated with the timber industry [2]. These wood residues, such as sawdust and planer shavings, are commonly used to produce non-structural composite panels like particle boards and medium-density fiberboards [3].

The construction industry is one of the largest consumers of natural resources and raw materials [4]. Additive manufacturing (AM) is a technique that can be used in the housing industry to reduce the environmental impact of construction [5]. While some research has been conducted on wood-based AM [6,7], most of these wood-based products use thermoplastic polymers (typically polylactic acid (PLA)) [8,9] as the matrix, which are extruded under hot conditions. Some previous work has explored utilizing thermoset composites as an alternative for additively manufactured wood composites in construction applications, commonly using sodium silicate (SS) or phenol formaldehyde (PF) or urea formaldehyde (UF) [10,11,12,13,14]. For wood-SS composites that were additively manufactured, the flexural strength was found to be between 10.47 and 7.23 MPa [10]. For a similar composite, a much broader range of 7.56 to 19.1 MPa was observed, based on post-AM curing conditions [12]. While these thermoset composites are suitable for AM, there is scope for further improving their mechanical properties.

The use of natural fibers in AM has shown potential to improve the mechanical, thermal, chemical, and morphological properties of the parts used in various fields, including civil, structural, and biomedical engineering [15]. Natural fibers, particularly hemp, are gaining attention as sustainable reinforcements in composite materials due to their beneficial properties like low density and high strength [16]. Studies have shown that hemp fiber reinforcement can significantly improve the mechanical properties of various polymer matrices [17,18,19,20,21]. The use of hemp fibers to reinforce thermosetting polymers has been investigated, with a main focus on epoxy resins. However, very few studies have investigated the reinforcement of wood–thermoset composites for additive manufacturing, which is a gap in the literature that this paper aims to address [22,23].

The impact of hemp fiber length on the strength of polybutylene succinate (PBS) based composites showed that using short (<2 mm) and long (<10 mm) hemp fibers improved Young’s modulus by up to 63% when combined with 5% overlap between adjacent print lines [24]. In polypropylene (PP)/Poly(styrene-b-(ethylene-co-butylene)-b-styrene) (SEBS)/hemp fiber composites, longer fibers (>2.5 mm) resulted in increased storage modulus by 82–90% compared to 58% for shorter fibers (~1.1 mm) [25]. Another study found that using 40% of 0.2 mm hemp fibers increased the strength of the PP composite, more than the longer 2 mm hemp fibers [26]. This was due to better dispersion and more homogenous composites with the shorter fibers.

Studies also tested how the quantity of hemp fiber affects the strength of the composite. A study on hemp fiber reinforcement of polylactic acid (PLA) filament for AM observed that, while 3 wt% hemp did not significantly improve Young’s modulus, 7.5 wt% increased it by 23%, and 10 wt% by 10.65% [27]. For hemp fiber reinforcement of silicone for AM, 15 wt% hemp fibers were noted as the upper limit, as higher amounts of hemp fiber lead to low ductility and an increase in viscosity [28]. For PP reinforced with 5, 10, and 20 wt% hemp fibers, the 5 wt% composite was observed to have the highest tensile strength, whereas the 20 wt% composite had the highest Young’s modulus [29].

A few studies report that adding high amounts of fibers causes an increase in viscosity, which limits the maximum fiber amounts that can be used for reinforcement [28,30]; however, the impact of fiber amounts on the resulting motor power affecting the extrudability of composites has not been studied. While viscosity may be useful in predicting extrudability, it is also important to study the impact of fiber amounts on the motor power, as insufficient motor power may hinder extrusion.

In this work, we study the effect of adding different fractions of hemp fibers of varying lengths to wood–thermoset (SS) composites for AM applications. The study focuses on the rheological behavior, extrudability, and bending/compression strength of the resulting composites. We hypothesize that the addition of hemp fibers will increase the flexural and compressive strengths of the wood–thermoset composites. We also anticipate that increasing hemp fiber content and varying fiber length will impact the rheological properties and extrudability of the composite for additive manufacturing applications.

## 2. Materials and Methods

### 2.1. Materials and Preparation

The composite formulations consisted of wood fibers, SS solution, and hemp fibers. Wood fibers in the form of sawmill residues were obtained from Plummer Forest Products (Post Falls, ID, USA). The residues were passed through a standard 40 mesh sieve (opening size: 0.381 mm). The sifted wood fiber was collected for use. Commercial SS solution (37%) received from ThermoFisher Scientific (Pittsburgh, PA, USA) was used as resin. ‘Degummed Hemp Sliver’ was purchased from Hemptraders.com.

The moisture content of wood and hemp fibers prior to extrusion and all testing was measured using a moisture analyzer ( Model HB43-S, Mettler Toledo, Columbus, OH, USA) in triplicate. The density of the fibers was measured using an ultra-pycnometer 1000 (Quantachrome, Boynton Beach, FL, USA) in triplicate.

**Figure 1:**
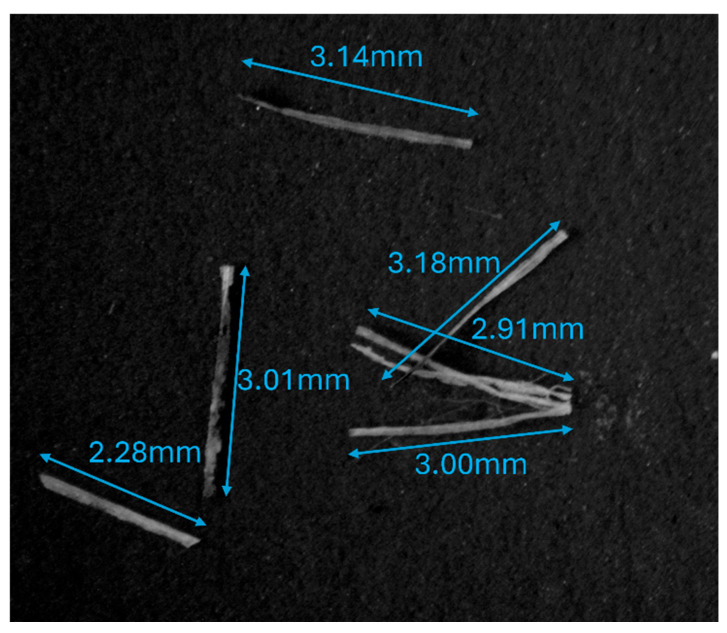
Hemp fiber length measurements for 3 mm cuts.

Hemp fibers were cut using an automatic textile ribbon cutter. This cutter allowed faster and more accurate cuts of various lengths (1, 3, 5 mm). Fiber lengths were measured using an AmScope Microscope with different magnification settings. A 5X magnification lens was used for the 1 mm fibers, while the 3 mm and 5 mm fibers were measured without any magnification lens. One hundred individual fibers of each intended length were measured to calculate the average length of the cut fibers. For the 1 mm fibers, the diameter was also measured, using the 5X magnification lens. For the 5 mm fibers, the fiber lengths were measured after preparing the composite to determine if the mixing and blending process, as described in Section 2.3, affected them. After preparation, the composite was dissolved in an aqueous solution to break it down, and hemp fibers for measurement were then collected using tweezers. The camera of the microscope (MU500-HS) was connected to a computer to capture images, and AmScope software (V4.12) was used to calibrate and measure the lengths of the fibers. Figure 1 shows an example of length measurement for 3 mm cuts.

Samples were generated by modifying hemp fiber length (1, 3, 5 mm), hemp fiber percentage (2.5, 5, 10 wt%), and SS percentage (45, 50, 55 wt%). The range of the factors was decided based on qualitative information from prior tests and observations. The SS amount was based on prior research by Carne et al. [10], where 50–55 wt% SS was found to be most suitable for AM. Preliminary tests showed that mixing and extrusion are more difficult when adding 15 wt% or more hemp fibers. Therefore, the hemp fiber amount was carefully selected to achieve effective reinforcement with minimal fiber addition (up to 10%). The lengths of the hemp fiber were similarly selected for ease of mixing, as longer fibers (>5 mm) tended to form clumps in preliminary tests.

The experiments were designed according to the Box–Behnken design for 3 factors with 3 continuous variables. A total of 13 different combinations were designed for these factors, and an additional control sample (50 wt% SS, 50 wt% wood fiber) was added for a total of 14 samples for extrusion and testing. Table 1 shows the experimental design. A Response Surface Analysis (including ANOVA) was conducted on Minitab (V22) at the 95% confidence interval to identify the significant variables. The main effects were plotted using Minitab (V22).

### 2.2. Rheology

Flow behavior for composite mixtures was determined using a DHR-2 Rheometer (TA Instruments, New Caste, DE, USA). Wood and hemp fiber were weighed according to the specified composition. SS was then added to the fiber mixture, manually mixed, and mixed using an electric herb grinder for 30 s. Specimens (2 mm × 25 mm Ø) were formed using a hydraulic press and a 25 mm pellet die. Frequency sweep experiments were performed using a 25 mm steel serrated parallel plate geometry from 0.01 Hz to 100 Hz at 25 °C with 0.1% strain and 3.0 N axial force control. Experiments were performed in triplicate, and the average complex viscosity (η*) at 1 Hz and 0.12 Hz for each composition was recorded.

The complex viscosity at 1 Hz frequency was used to align with prior experiments conducted in previously published work [10,11,12]. The complex viscosity at 0.12 Hz is also reported, to better align with the shear rates expected within the extruder, according to Equation [31]:

γ ˙ = πDN/60 h(1)

where γ ˙ is the shear rate in the single screw extruder, D is the diameter of the barrel, in mm, N is the speed of the motor, in rpm, and h is the channel depth, in mm.

Using Equation (1) for the extruder, we calculate the shear rate to be 3.24 s^−1^. We then used this shear rate to calculate the frequency in the rheometer to be 0.12 Hz.

### 2.3. Extrusion and Specimen Preparation

**Figure 2:**
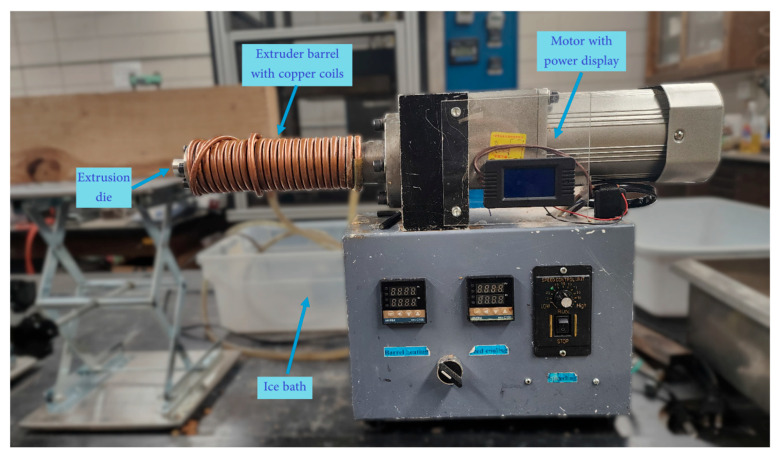
Extruder setup.

Sixty grams (dry basis) of each composition shown in Table 1 were prepared. Varying amounts of wood fiber (based on the formulation) were weighed and added to a large blender (Waring 700B, Waring commercial, CT, USA). Then, hemp fibers were added and blended for approximately 2 min to make a fiber composite mixture. The SS solution was then added to the mixture. The entire composite mixture was then blended for an additional 2 min. The composite samples were prepared using a commercial single screw extruder (Robot Digg, Shanghai, China). For this study, a die with a diameter of 9.4 mm was used with a no-compression auger. The motor, which was rated to a maximum of 300 W, was set at 13 rpm. A cooling coil (copper tube of 6 mm diameter) was installed on the barrel to prevent overheating. An ice-water bath fed the cooling system (360 L/h) and was replaced as the ice melted to maintain a constant temperature throughout the experiments. The extruder setup is shown in Figure 2.

This mixture was hand-fed continuously into the extruder barrel. A cardboard edge protector was used to collect the extruded composite from the die. Power consumption was monitored continuously during extrusion, with the maximum observed power recorded (rounded up to the nearest 5 W). The overall difficulty of feeding the materials was evaluated, as some compositions showed significantly greater resistance to flow through the feeding slot. During hand-feeding into the barrel, the difficulty was qualitatively assessed and classified as easy, medium, or hard based on the force needed to push the mixture through. The total extrusion time, defined as the duration from the start of feeding until all sixty grams of the mixture exited the die, was recorded and rounded to the nearest 5 min increment. The length of extrudate in a single batch was roughly 1 m.

After extrusion, the extrudate was placed with the cardboard base in an oven at 105 °C for 24 hr for curing. The cardboard base allowed more uniform curing as it allows moisture to escape from the bottom of the extrudate. After 24 hr, the material was removed and allowed to cool to room temperature. Specimens for flexural testing, compression testing, and density measurements were made from this cured material. All materials were placed in sealable polyethylene bags at the beginning of the study to prevent any change in moisture content during the duration of the test. Specimens once oven-cured were similarly placed in sealable polyethylene bags after extruding and weighing.

### 2.4. Flexural/Bending Test

Five specimens of 170 mm length were cut from the extrudate using a miter saw. Three-point flexure testing was performed according to ASTM D790 [32] (with modifications for a cylindrical specimen, according to ASTM D6109 [33]) using Mecmesin MutiTest-dV 2.5 (Sterling, Virginia, USA). The test span was set to 16 times the diameter of the specimens (150 mm). The test was performed at a crosshead speed of 4 mm/min, and the data was exported into a raw file in CSV format for analysis.

### 2.5. Compression Test

Five 18.8 mm specimens were cut from the extrudate using a miter saw. Compression tests were performed according to ASTM D695 [34] (crosshead speed of 0.1 mm/min) using an Instron 5500R universal testing machine (Norwood, MA, USA) with a 2.5 kN loadcell. Experimental data and results were collected from the Bluehill universal material testing software (V4.44).

### 2.6. 3D Printing

**Figure 3:**
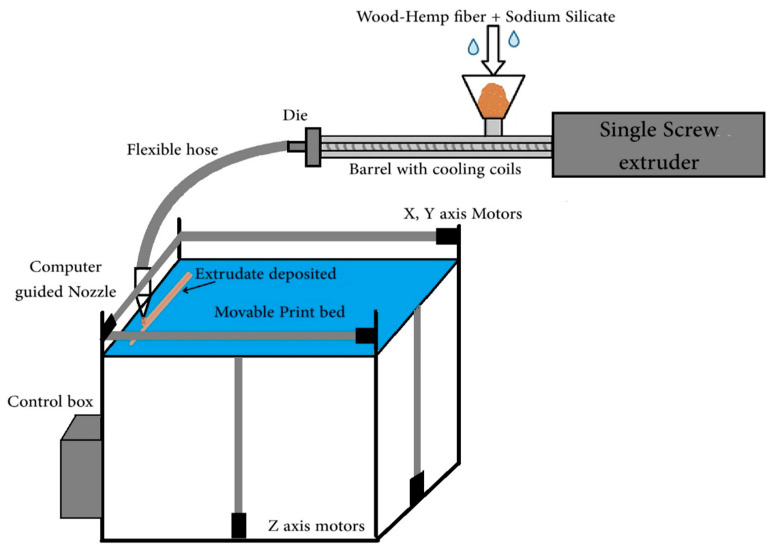
Schematic diagram of the additive manufacturing principle of hemp fiber-reinforced materials.

A formulation with hemp fiber was selected based on the results of the tests conducted in this study. The materials were mixed in the same order as described in Section 2.3 in a high-shear-rate food processor and then fed manually into the barrel of a custom-built extruder (3-horsepower motor). A total of 400 g of fiber (360 g of wood fiber, 40 g of hemp fiber) was used along with an equal amount of SS to extrude and print 2 layers, with total dimensions of 150 mm length, 40 mm width, and 30 mm height. A simple 2-layer G-code was written and imported into Pronterface software (V2.1) on a computer that was connected to a custom-built 3D printer [10]. The overall 3D printing process closely followed the methodology described by Rober Carne et al. [10]. Similar to the extrudates, the printed sample was cured in the oven at 105 °C for 24 h. A schematic diagram of the additive manufacturing principle of hemp fiber-reinforced materials is shown below in Figure 3.

## 3. Results and Discussion

### 3.1. Density, Moisture Content, and Hemp Fiber Length

The density of wood fibers and hemp fibers was measured to be 1.48 ± 0.01 g/mL and 1.58 ± 0.01 g/mL, respectively. The moisture content of the wood fibers and hemp fibers was measured to be 6.81 ± 0.18% and 7.35 ± 0.07%, respectively. While prior research showed that the moisture content of materials can significantly impact the mechanical properties of composites [10,12], this analysis is beyond the scope of the current study. However, the moisture content of the materials is noted for future analysis.

**Figure 4:**
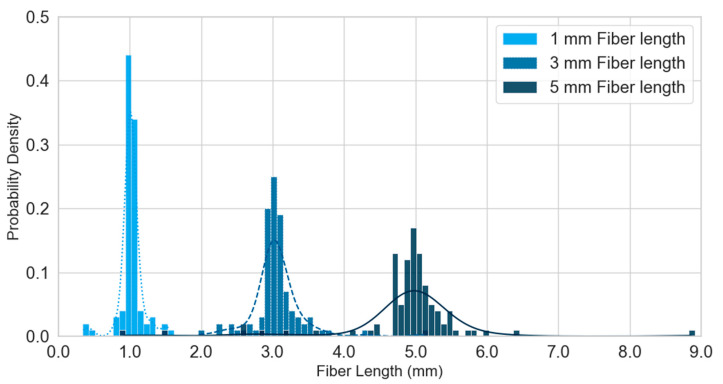
Probability distribution of cut hemp fibers.

The precision of the hemp fiber cutting process was evaluated, yielding average lengths of 1.02 mm, 3.06 mm, and 4.88 mm for the nominal 1 mm, 3 mm, and 5 mm settings, respectively. The coefficient of variance was lowest for the 1 mm fibers (0.12) and progressively increased for the 3 mm (0.16) and 5 mm (0.18) fibers, suggesting that achieving highly uniform lengths becomes more challenging with increasing fiber length. This variability in fiber length, particularly for longer fibers, is a factor that could influence composite properties and processing behavior. Figure 4 shows the probability distribution for the chopped hemp fiber lengths. The fiber diameter was measured to be an average of 66 µm with a standard deviation of 49 µm. The aspect ratio for 1 mm fibers is an average of 15.5, for 3 mm fibers, it is 46.4, and for 5 mm fibers, the aspect ratio is 73.9. According to the Kelly–Tyson model, longer fibers should increase the flexural strength of the composite more than shorter fibers [35]. The average length of the 5 mm fibers measured after composite preparation was 4.98 mm, and the coefficient of variance was 0.14. There was no statistically significant difference between the fiber lengths before and after composite preparation (*p* > 0.3). Thus, the composite preparation method did not cause a significant reduction in length.

### 3.2. Rheology

Rheology data were collected, and the complex viscosity at 0.12 Hz and 1 Hz for each formulation was imported into Minitab for analysis. Analysis of Variance (ANOVA) indicated that at both 1 Hz and 0.12 Hz, viscosity was influenced by the amount of SS used (*p* < 0.02), with viscosity increasing as the SS amount was increased. This occurs because a higher SS amount results in higher liquid content, increasing viscosity. Similarly, higher solid content led to lower viscosity. A simple analogy is a rotating parallel plate encountering resistance against a viscous liquid surface, yet sliding easily over a granular solid like sand.

Increasing hemp fiber length and quantity both reduced the complex viscosity; however, the effect was not statistically significant at a 95% confidence level (*p* > 0.1). The percentage contribution of SS% towards complex viscosity is 68.03%. Other factors contribute less than 10% each.

**Figure 5:**
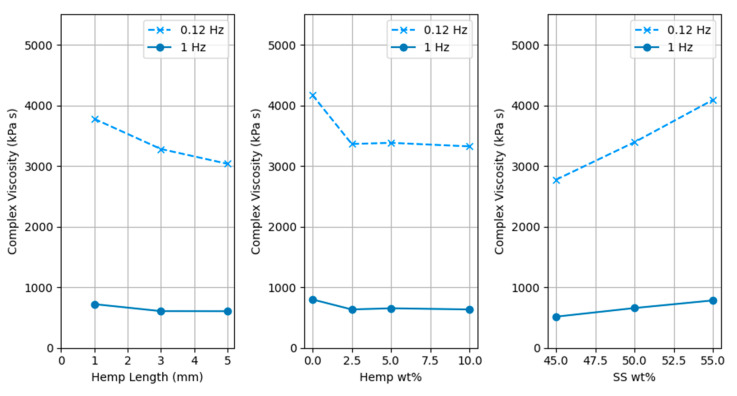
Main effects for complex viscosity at 0.12 and 1 Hz.

It was also observed that the complex viscosity at 0.12 Hz is about 5.24 times higher than at 1 Hz. This is expected as wood-SS composites have shear-thinning behavior, and at higher frequency, complex viscosity reduces [11]. Figure 5 shows the main effects for complex viscosity.

It is important to note that the specimens prepared had a thickness of 2 mm, and the data for specimens with longer hemp fibers (3, 5 mm) might not perfectly represent the true bulk rheological properties due to wall effects or fiber bridging. However, this methodology was selected despite this drawback in order to align with other experiments in previously published work [10,11,12]. This potential inaccuracy should be borne in mind when interpreting the non-significant effect of fiber length and quantity on viscosity. Despite this, the strong influence of SS content on viscosity remains a robust finding.

### 3.3. Extrusion and Power

The baseline motor power of the extruder measured without any extrudate was 60 W. Power increased gradually as the mixture was added to the extruder barrel. In some cases, power reached a maximum value. This peak in power corresponded to relatively slow extrusion observed at the die exit. Subsequently, the power decreased as the material resumed flowing normally. Maximum power on the motor was recorded during the extrusion, rounded up to the next multiple of 5.

The data was statistically analyzed using response surface design [36]. Statistical analysis of the maximum motor power recorded during extrusion revealed that the SS amount was a highly significant factor (*p* < 0.01), contributing a substantial 78.59% to the observed motor power variation. In contrast, the hemp fiber amount, hemp fiber length, and their interactions were found to be statistically insignificant (*p* > 0.3), contributing less than 10% each.

A key finding was that higher percentages of SS content led to a reduction in the maximum power exerted on the motor during extrusion. While higher SS content was shown to increase the complex viscosity of the material as measured by the rheometer, it simultaneously reduced the motor power required for extrusion. This contrasts with what is typically observed and expected, that, as viscosity is reduced, resistance is reduced, thereby reducing the power required to move the material [37]. This could be explained by the different operating conditions and material properties. Higher solid content at lower SS levels resulted in lower viscosity under parallel plate rheometry, as solids offer less resistance to plate rotation. Whereas higher solid content increases the friction and resistance in a high-pressure, confined barrel with continuous flow. This increased friction increases the motor power required despite the lower viscosity observed in the parallel plate condition.

**Figure 6:**
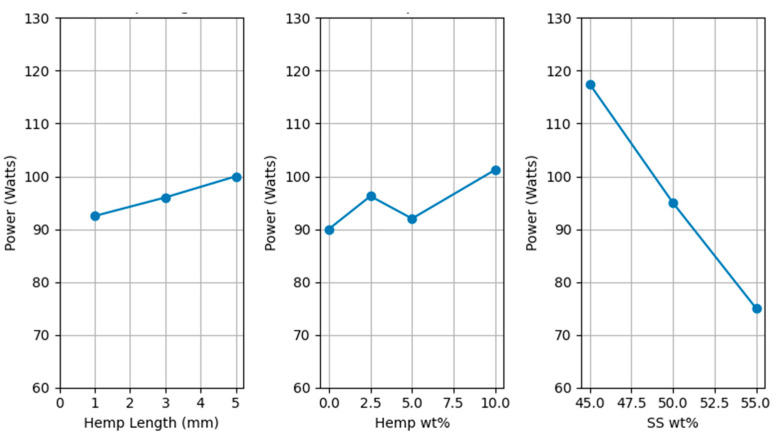
Main effects for motor power for extrusion.

Figure 6 shows the main effects plot for motor power. The ordinate (y-axis) of the plot is truncated from the baseline power (60 W). It shows that the maximum power exerted on the motor reduces with a higher percentage of SS. As the motor is rated at 300 W, all these combinations were extruded. However, it was qualitatively noted that the motor temperature increased significantly after 30 min of extrusion. This suggests that for longer extrusion runs, the maximum power drawn by the motor, even if within its rated capacity, could lead to thermal management challenges. Furthermore, lower SS amounts were qualitatively associated with greater difficulty in feeding the material into the extruder and longer overall extrusion times. These qualitative observations have crucial practical implications for process optimization and scalability, underscoring the importance of SS content. Extrusion times and feeding difficulties are summarized in Table 2.

### 3.4. Flexural/Bending Test

The flexural strength of extruded specimens cured at 105 °C was measured. The data were statistically analyzed using response surface design. ANOVA showed that strength depends on hemp fiber amount (*p* < 0.03), with flexure strength increasing with increasing hemp fiber amount. Other factors and interactions were not significant at 95% confidence (*p* > 0.07). The percentage contribution of hemp fiber amount on flexural strength is 54.12%. Other factors contribute less than 10% each.

The average bending strength without any hemp fiber (50 wt% wood, 50 wt% SS) was 16.64 MPa. The bending strength increased by 9.89%, 31.2%, and 46.9% with 2.5 wt%, 5 wt%, and 10 wt% hemp fiber, respectively. These findings align with prior research on Wood-SS composites (50 wt% wood, 50 wt% SS), where reported flexural strengths range from 7.2 to 23.5 MPa under various curing and processing conditions [10,11,12,38]. It is worth noting that slower curing conditions at lower temperatures (e.g., 60°C for 72 h) have been shown to yield higher flexural strengths in similar systems [12].

**Figure 7:**
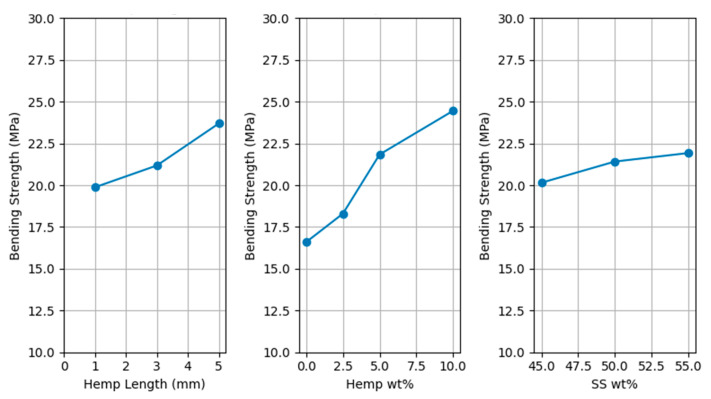
Main effects for bending strength.

Samples with longer hemp fibers appear to have higher strength. The SS amount appears to have minimal effect on bending strength, with 55 wt% SS having slightly higher mean bending strength. This indicates that while a trend might be visually discernible, the study’s design or sample size may not have been sufficient to statistically confirm these effects, given the inherent variability. Additionally, longer fibers were qualitatively linked to increased challenges in composite mixing and feeding into the barrel, which may offset the theoretical benefits of enhanced load transfer associated with longer fibers. Figure 7 shows the main effects plot for bending strength.

### 3.5. Compression Test

The compression strength of all the specimens was measured and analyzed. The compression strength of the sample without hemp fiber (50 wt% wood, 50 wt% SS) was 14.8 MPa. With the addition of hemp fiber, the compression strength increased significantly to 20.1 MPa (5 wt% hemp fiber), representing a 35.6% increase; this result was also statistically significant (*p* < 0.04). This substantial enhancement underscores the effectiveness of hemp fibers as a reinforcing agent in these composites.

While a trend of increasing compression strength was observed with longer fibers and higher SS content, ANOVA revealed that these factors were not statistically significant at the 95% confidence level (*p* > 0.1). The percentage contribution of the hemp fiber amount on compression strength is 37.05%. The contribution of hemp fiber length to compression strength is 16.0%. Other factors contribute less than 10% each.

**Figure 8:**
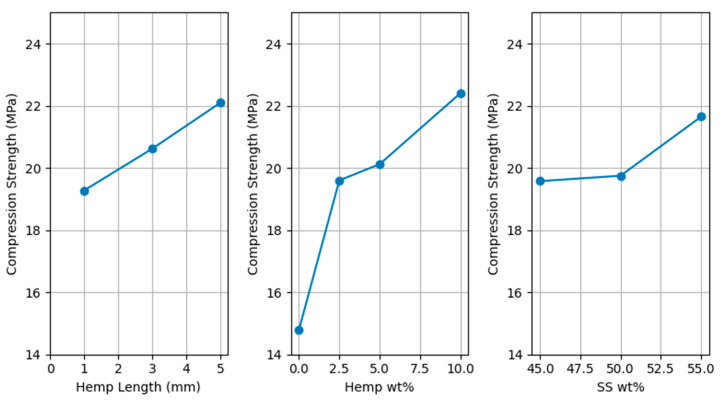
Main effects for compression strength.

The main effects for compression strength are shown in Figure 8.

### 3.6. 3D Printing

The successful 3D printing of a two-layer sample using a formulation of 45 wt% wood fiber, 5 wt% hemp fiber of 3 mm length, and 50 wt% SS serves as a critical proof of concept for the material’s applicability in additive manufacturing. This formulation was selected because at 5 wt% of hemp fiber, bending and compression strength were increased greatly, with diminishing returns at 10 wt%. While 5 mm hemp fibers appeared to produce stronger composites, the effect was not statistically significant, and formulations with 3 mm hemp fibers were easier to mix and feed. The formulations with 3 mm hemp fibers were also associated with lower motor power during extrusion. A 50 wt% SS was selected for the test print to directly compare with prior research [10].

**Figure 9:**
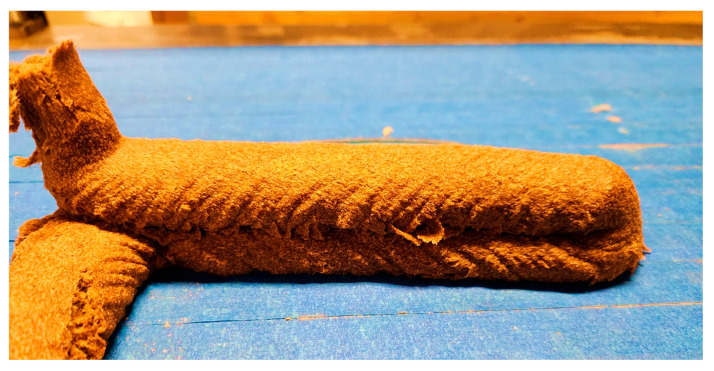
Sample print with 5 wt% hemp fiber.

The printed part is shown in Figure 9. The printed part has a thickness of 30 ± 1 mm and a width of 40 ± 5 mm, showing its geometric uniformity. The flexural strength of the two-layer sample was found to be 13.24 MPa, representing a 26.5% increase over previously reported values [10]. It should be noted that this is based on a single sample, so further testing is required to confirm the dimensional and flexural properties.

## 4. Conclusions

This study highlights the potential of hemp fiber reinforcement in improving the mechanical properties of wood-SS composites. Particularly, the addition of just 5 wt% hemp fibers resulted in a significant improvement of 30–35% in bending and compression strength. While further increases in hemp fiber amount continued to increase strength, diminishing returns were observed, suggesting an efficient utilization of reinforcement at the 5 wt% level.

The extrusion process showed an increase in power consumption with longer and increasing concentration of hemp fibers, but this effect was not statistically significant and could be mitigated by increasing the SS content to 55 wt%. Hemp fiber length has a positive effect on bending and compression strength, although the effect is not statistically significant. Considering this lack of statistical significance and the observed increased motor power required for longer fibers, a hemp fiber length of 1–3 mm is recommended. This recommendation synthesizes scientific findings with practical engineering feasibility, offering a balanced approach that optimizes mechanical performance while maintaining efficient processability and lower energy demands.

Finally, the study successfully demonstrated the feasibility of 3D printing wood-SS composites reinforced with 5 wt% of 3 mm length hemp fibers. This serves as a crucial proof of concept, indicating the potential for these materials to be used in manufacturing structures with improved mechanical properties. This work directly addresses a significant gap in the literature regarding the reinforcement of wood–thermoset composites for additive manufacturing, providing foundational data and practical insights for the development of more sustainable and structurally enhanced materials for construction and other applications.

This study has several limitations that provide clear directions for future research. To validate the material for AM applications, an investigation into the dimensional stability of extruded specimens is needed, both before and after curing. The shear strength between the matrix and hemp fibers should also be studied. Effects of moisture and ambient conditions during extrusion need further understanding. While the two-layer 3D-printed specimen serves as a proof of concept, a larger number of printed specimens should be tested to confirm the mechanical properties. Interlayer bond strength also needs to be evaluated. Further analysis is required to identify and optimize the composite formulation, including a cost–benefit analysis. The development of capabilities to print more complex geometries will also be a necessary next step.

## Figures and Tables

**Table 1:** Formulations for experiments.

Formulation	Hemp Fiber Length (mm)	Hemp wt%	SS wt%	Wood Fiber wt%
1	3	2.5	45	52.5
2	3	2.5	55	42.5
3	3	10	45	45
4	3	10	55	35
5	3	5	50	45
6	1	2.5	50	47.5
7	1	10	50	40
8	1	5	45	50
9	1	5	55	40
10	5	2.5	50	47.5
11	5	10	50	40
12	5	5	45	50
13	5	5	55	40
14	0	0	50	50

**Table 2:** Summary of results.

Hemp Length (mm)	Hemp wt%	SS wt%	Power (Watts)	Feeding Difficulty	Extrusion Time (min)	Complex Viscosity at 1 Hz (kPa s)	Bending Strength (MPa)	Compression Strength (MPa)
3	2.5	45	125	Hard	25	519	15.5	18.4
3	2.5	55	75	Easy	20	710	18.9	19.9
3	10	45	125	Hard	40	50	22.7	23.2
3	10	55	70	Easy	20	744	25.4	22.2
3	5	50	85	Medium	25	55	23.4	19.4
1	2.5	50	90	Easy	30	673	19.1	18.2
1	10	50	95	Medium	35	671	23.1	20.9
1	5	45	115	Medium	30	601	17.0	16.6
1	5	55	70	Easy	25	940	20.3	21.4
5	2.5	50	95	Easy	20	632	19.7	21.9
5	10	50	115	Hard	45	614	26.6	23.3
5	5	45	105	Hard	45	433	25.4	20.1
5	5	55	85	Medium	30	739	23.1	23.1
0	0	50	90	Easy	25	799	16.6	14.8

## Data Availability

The original contributions presented in this study are included in the article. Further inquiries can be directed to the corresponding author(s).
